# p73 regulates basal and starvation-induced liver metabolism *in vivo*

**DOI:** 10.18632/oncotarget.5090

**Published:** 2015-09-07

**Authors:** Zhaoyue He, Massimiliano Agostini, He Liu, Gerry Melino, Hans-Uwe Simon

**Affiliations:** ^1^ Institute of Pharmacology, University of Bern, Bern, Switzerland; ^2^ Medical Research Council, Toxicology Unit, Leicester, United Kingdom; ^3^ Department of Experimental Medicine and Surgery, University of Rome “Tor Vergata”, Rome, Italy

**Keywords:** p73, starvation, metabolism, liver, autophagy

## Abstract

As a member of the p53 gene family, p73 regulates cell cycle arrest, apoptosis, neurogenesis, immunity and inflammation. Recently, p73 has been shown to transcriptionally regulate selective metabolic enzymes, such as cytochrome c oxidase subunit IV isoform 1, glucose 6-phosphate dehydrogenase and glutaminase-2, resulting in significant effects on metabolism, including hepatocellular lipid metabolism, glutathione homeostasis and the pentose phosphate pathway. In order to further investigate the metabolic effect of p73, here, we compared the global metabolic profile of livers from p73 knockout and wild-type mice under both control and starvation conditions. Our results show that the depletion of all p73 isoforms cause altered lysine metabolism and glycolysis, distinct patterns for glutathione synthesis and Krebs cycle, as well as an elevated pentose phosphate pathway and abnormal lipid accumulation. These results indicate that p73 regulates basal and starvation-induced fuel metabolism in the liver, a finding that is likely to be highly relevant for metabolism-associated disorders, such as diabetes and cancer.

## INTRODUCTION

The transcription factor p73 belongs to the p53 gene family and shares structural and functional homology to the tumor suppressor p53 [1–9]. Due to alternative promoters near the *N*-terminus, p73 is mainly transcribed in two types of isoforms, the transcriptionally active TAp73 and the dominant negative ΔNp73 [10, 11]. TAp73 isoforms act as tumor suppressors by inducing apoptosis, cell cycle arrest and maintaining genomic stability, whereas ΔNp73 variants counteract the tumor-suppressor activity of p73 and p53 by directly binding to the p53 response element or by the formation of inactive oligomers [12–15]. p73 knockout (KO) mice exhibit severe neurological and immunological defects due to the absence of all p73 isoforms [16]. Mice lacking TAp73 show infertility, formation of spontaneous tumors and elevated sensitivity to chemical carcinogens, highlighting the tumor suppressive function of TAp73 [17]. Conversely, ΔNp73 KO mice are fertile with signs of moderate neurodegeneration and, interestingly, diminished tumor development [18]. However, the *TP73* gene is rarely mutated or deleted in human cancer, suggesting a more complicated scenario for the role of p73 in cancer [1, 19]. Accordingly, the balance between the TAp73 and the ΔNp73 isoforms dictates the fate of the individual cell, and the outcome of the tumor.

Besides its tumor-suppressive role, TAp73 has been shown to be capable of regulating metabolism [20, 21]. For instance, overexpression of TAp73α in Saos-2 cells increased mitochondrial activity to boost glutathione homeostasis and to elevate the activity of the pentose phosphate pathway (PPP) [22]. TAp73 is able to directly regulate the transcription of Cox4i1, affecting the function of complex IV in mitochondria and resulting in a redox imbalance that contributes to premature senescence and aging [23, 24]. TAp73 has also a direct effect on the serine/glycine biosynthesis. Indeed, metabolic profiling of human cancer cells revealed that TAp73 regulates serine biosynthesis by transcriptional control of glutaminase-2 (GLS-2), an enzyme that supports glutathione synthesis and enables an effective compensation for an excessive oxidative response [25, 26]. As a consequence, TAp73 has a profound effect on cellular metabolism in a manner similar, but distinct to p53 and p63 [27, 28]. In addition to this finding, TAp73 enhances the PPP flux via transcriptional activation of a rate-limiting enzyme in PPP, the glucose-6-phosphate dehydrogenase (G6PD) [29]. These findings are consistent with the current understanding on the role of TAp73 as a potent tumor suppressor [30] indicating that TAp73 mounts an anti-senescence metabolic response [31]. Accordingly, recently published metabolomics studies of cancer cells revealed that TAp73 promotes anabolism to counteract cellular senescence rather than to support proliferation [32]. Hence, the newly revealed, exciting role of p73 in metabolism is complex and requires more follow-up *in vitro* and *in vivo* studies.

Our previous study showed that p73 regulates autophagy by transcriptional activation of autophagy-related protein 5 (ATG5) and pointed out the importance of p73 in the hepatocellular lipid metabolism [33]. To investigate the role of p73 in the hepatocellular metabolism *in vivo*, we performed metabolic profiling of the livers from both WT and the full p73 KO mice under both control and starvation conditions. Our data show that p73 depletion results in altered metabolic pathways, including lysine and methionine metabolism, glutathione homeostasis, PPP, Krebs cycle, and lipid metabolism. In contrast to previously published work [29], we observed an increased, instead of decreased, PPP flux in the p73 KO mice, implying the existence of other transcriptional factors regulating the G6PD expression.

## RESULTS

### Different metabolic profiles in WT and p73 KO mice

**Figure 1:**
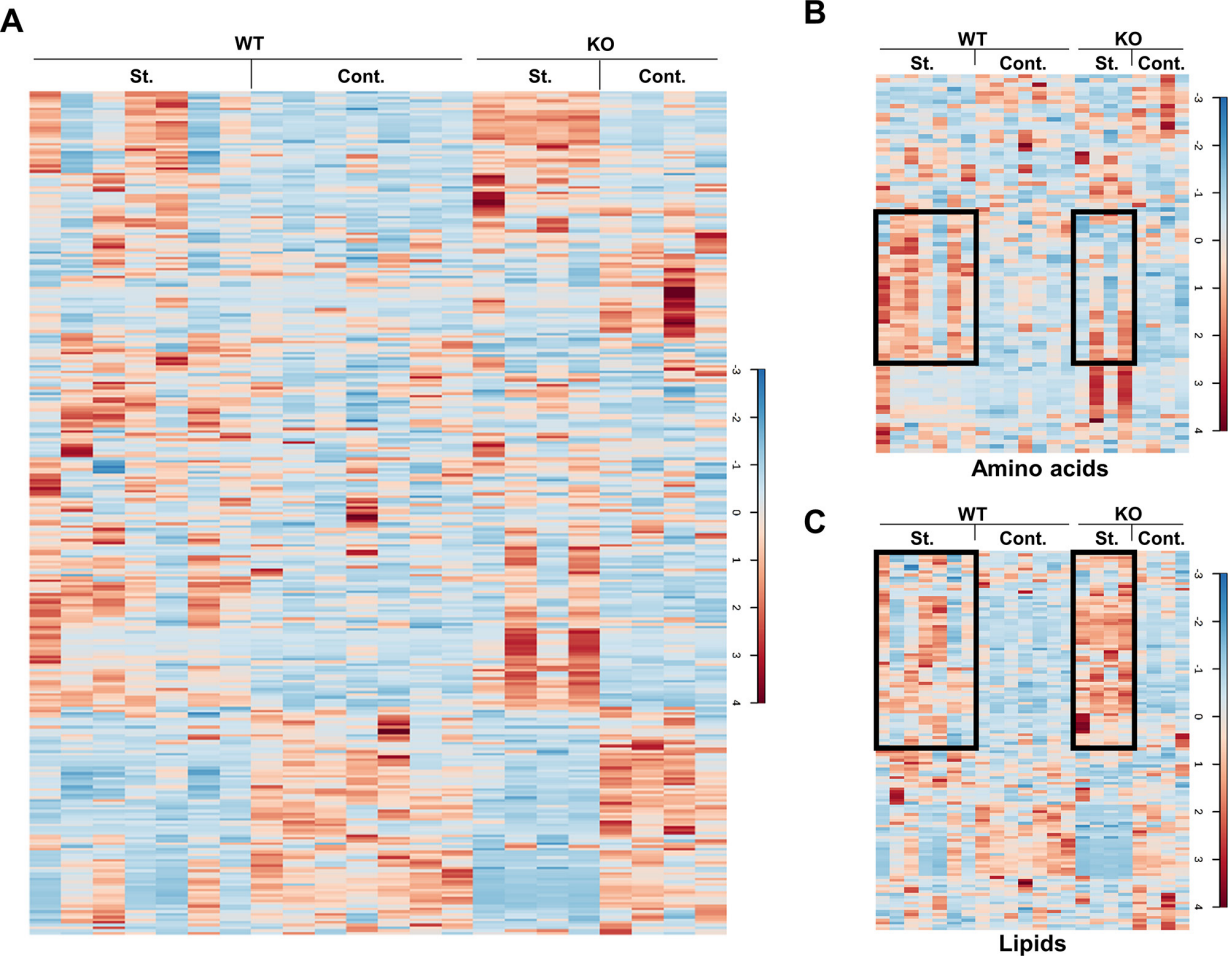
Heat maps of the metabolites Liver samples of the control and starved mice were extracted and prepared for the MS analysis. The levels of the total 347 metabolites are shown as a heat map in **A.** Heat maps of the metabolite subgroups, amino acids and lipids, are shown in **B.** and **C.** The black boxes indicate the extensive changes of the metabolites under different conditions. St.: starvation; Cont.: control.

To investigate the role of p73 in starvation-induced metabolic changes, liver samples from both control and starved WT and p73 KO mice were collected and prepared for global biochemical profile by GC/MS or LC/MS. For each group, 7 individual mice for the wild genotype and 4 individual mice for the p73 KO genotype were analyzed separately. Mice were all littermates, untreated or starved for 24 hours before being sacrificed. All these biological replicates were immediately stored at −80°C until extracted and prepared for analysis. Following statistical analysis by 2-way ANOVA and ANOVA contrasts, biochemical metabolites showing statistical significance (*p* ≤ 0.05), as well as those approaching significance (0.05 < *p* < 0.1), were tabulated and summarized in Table 1. In total, 347 compounds of known identity were identified in this study (Supplementary Table S1). Heat maps of total 347 metabolites and subgroups are shown in Figure 1 and Supplementary Figure S1. As expected, starvation dramatically altered the metabolism as indicated for 184 compounds, which exhibited significant change. Overall, fasting for 24 hours induced a metabolic program consistent with stimulation of autophagy as evidenced by increased amino acids, depletion of glycogen, and a shift from carbohydrate to lipid metabolism, including elevated ketogenesis. A noticeable number of metabolites (71) showing significant genotype-based differences were detected between the WT and p73 KO mice, underlining the importance of p73 both for basal and starvation-induced liver metabolism.

**Table 1:** Statistical summary of significant alterations of the metabolites

*ANOVA Contrasts*	Starved/Control		p73 KO/WT	
	WT	p73 KO	Control	Starved
Total biochemicals *p* ≤ 0.05	118	131	42	37
Biochemicals (increased/decreased)	68|50	58|73	31|11	18|19
Total biochemicals 0.05 < *p* < 0.10	38	34	25	38
Biochemicals (increased/decreased)	25|13	20|14	14|11	14|24
***Two-Way ANOVA***	**Genotype Main Effect**	**Treatment Main Effect**	**Genotype:Treatment Interaction**	
Total biochemicals *p* ≤ 0.05	71	184	34	
Total biochemicals 0.05 < *p* < 0.10	35	19	32	

Contrasts resulting from 2-way ANOVA shown in the upper table were used to identify the metabolites that differed significantly between experimental groups. The lower table shows the numbers of significantly changed metabolites in consideration of genotype, treatment, and interaction.

Starvation induces a metabolic switch from carbohydrate to lipid metabolism [34]. Several metabolic pathways are normally upregulated, including glycogenolysis, gluconeogenesis, lipolysis, and ketogenesis. In our study, depletion of hepatic glycogen was observed to a similar extent in both WT and p73 KO mice after starvation, as evidenced by significant reductions in the glycogen degradation products, such as maltose, maltotriose, maltotetraose, maltopentaose, and maltohexaose suggesting that p73 is not involved in glycogenolysis (Supplementary Table S1). Starvation for 24 hours induced significant elevations in many free amino acids, which is likely attributable to increased skeletal muscle protein degradation and subsequent uptake of amino acids by the liver for entry into the gluconeogenic pathway. Interestingly, starvation-induced effects on protein metabolism may be less pronounced in p73 KO mice, as many amino acids were elevated to a lesser degree as compared to wild-type mice. This is, presumably owing, at least partially, to the deficiency of autophagy (Supplementary Figure S1) [33]. In addition, p73 KO mouse livers showed an increased lipid content upon starvation compared to the WT mice (Figure 1C). These data suggest that p73 depletion may cause defective lipid degradation or utilization, resulting from defective autophagy or a decreased rate of β-oxidation. Overall, starvation for 24 hours induced a metabolic switch in both WT and p73 KO mice and p73 KO mice exhibited genotype-based differences in several metabolic pathways compared to those of WT mice, which are presented below.

### Altered lysine and methionine metabolism and glutathione synthesis in p73 KO mice

**Figure 2:**
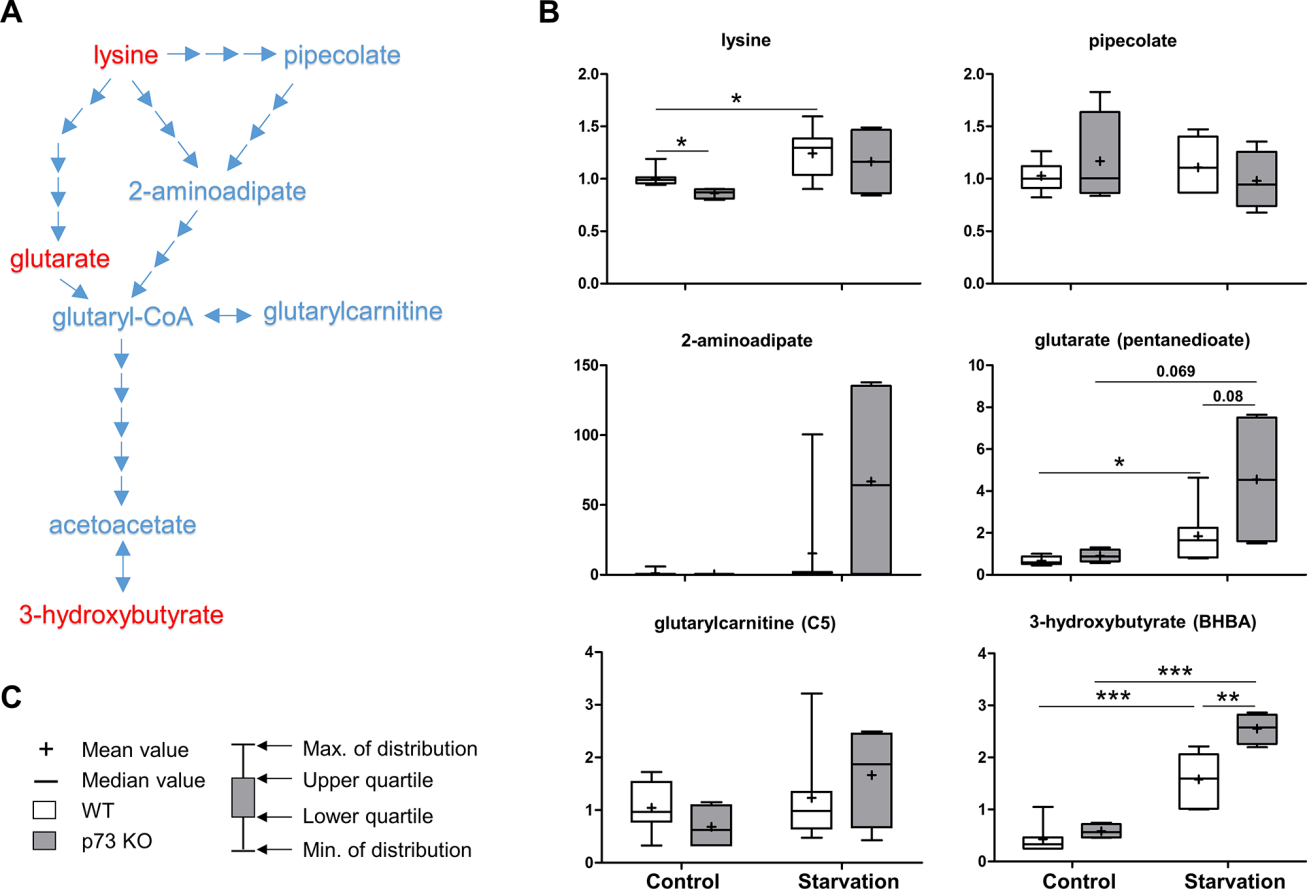
Lysine metabolism Lysine is an essential amino acid that can contribute to energy production through conversion to the ketone bodies, acetoacetate and 3-hydroxybutyrate. A schematic diagram for lysine metabolism is shown in **A.** Levels of the metabolites found in this pathway are shown in **B.** *P* values were calculated via 2-way ANOVA contrasts test. * < 0.05; ** < 0.01; *** < 0.001; *p* values between 0.05 and 0.1 are shown as numbers. **C.** Legends description of box-and-whiskers plot. *Y*-axis: relative amount of metabolite. Metabolites showing significant changes are highlighted in red.

Lysine belongs to the ketogenic amino acids that are able to produce ketone bodies in the liver under starvation conditions [35]. Ketone bodies include acetone, as well as acetoacetate and 3-hydroxybutyrate, both of which serve as important fuels for the brain. They are produced by breakdown of amino acids or fatty acids during prolonged starvation with a low glucose level in the blood. Here, intermediates of the lysine metabolism pathway, especially 2-aminoadipate and glutarate were much more elevated in p73 KO mice compared to those of WT mice upon starvation. Accordingly, the ketone body, 3-hydroxybutyrate also showed increased levels in the p73 KO mice under starvation condition (Figure 2). These findings thus suggest that the expression of enzymes involved in the lysine metabolism may be affected by p73 and the regulation of ketogenesis may differ between WT and p73 KO mice under starvation condition.

**Figure 3:**
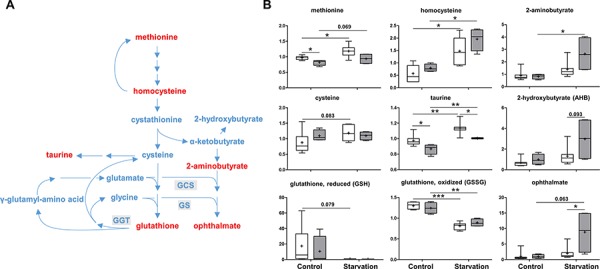
Methionine metabolism and glutathione synthesis As an important antioxidant, glutathione is synthesized from glycine, glutamate and methionine-derived cysteine. **A.** A schematic diagram of methionine metabolism and the glutathione synthesis pathway. Levels of the metabolites found in this pathway are shown in **B.** GCS: γ-glutamylcysteine synthetase, GS: glutathione synthetase, GGT: γ-glutamyltranspeptidase. *P* values were calculated via 2-way ANOVA contrasts test. * < 0.05; ** < 0.01; *** < 0.001; *p* values between 0.05 and 0.1 are shown as numbers. *Y*-axis: relative amount of metabolite. Legends descriptions are the same as in Figure 2C. Metabolites showing significant changes are highlighted in red.

As an important antioxidant, reduced glutathione (GSH) prevents cellular damage caused by reactive oxygen species. Once oxidized, GSH readily reacts with another reactive glutathione to form glutathione disulfide (GSSG) which is then reduced to GSH using NADPH. Glutathione can be synthesized from amino acids, including glycine, glutamate and methionine-derived cysteine. Under control conditions, methionine levels were reduced while increases in homocysteine and cysteine were observed in p73 KO mice as compared to those of WT mice. Together with a non-significant decrease in GSH and similar levels of GSSG, these findings may be suggestive of an attempt to increase glutathione synthesis, possibly in response to greater glutathione utilization in p73 KO mice (Figure 3). Under starvation conditions, an apparent increase in glutathione synthesis accompanied by depletions in both reduced and oxidized glutathione and a significant elevation in the biochemical marker of oxidative stress, cysteine-glutathione disulfide (Supplementary Table S1). These changes are consistent with the oxidative insult associated with increased mitochondrial β-oxidation of fatty acids during starvation. Interestingly, the subpathway producing ophthalmate (produced when 2-aminobutyrate is substituted for cysteine and is synthesized by the same enzymes as glutathione), a glutathione-like compound was much more activated in the p73 KO mice than that in the WT mice under starvation conditions as shown by increased levels of 2-aminobutyrate, 2-hydroxybutyrate and ophthalmate (Figure 3). These findings indicate that the enzyme involved in the ophthalmate subpathway is possibly regulated by p73. Hence, p73 KO mouse livers might exhibit a more oxidative environment compared to WT mouse livers.

### Differential glycolysis and elevated PPP in p73 KO mice

**Figure 4:**
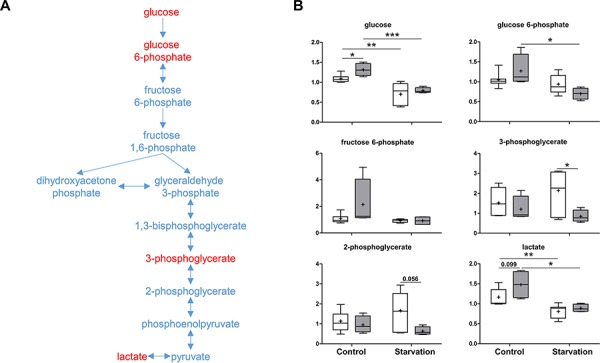
Glycolysis As a major catabolic pathway, glycolysis produces ATP by converting glucose to pyruvate, which can then be transferred to the mitochondria to enter the Krebs cycle for further energy production. **A.** A schematic diagram for glycolysis. Levels of the metabolites found in this pathway are shown in **B.** *P* values were calculated via 2-way ANOVA contrasts test. * < 0.05; ** < 0.01; *** < 0.001; *p* values between 0.05 and 0.1 are shown as numbers. *Y*-axis: relative amount of metabolite. Legends descriptions are the same as in Figure 2C. Metabolites showing significant changes are highlighted in red.

As a central metabolite, glucose can be oxidized to pyruvate via glycolysis or oxidized via the PPP to yield ribose 5-phosphate for nucleic acid synthesis and NADPH for reductive processes. In the p73 KO mice, increases in glucose, glucose 6-phosphate, and fructose 6-phosphate were detected under control conditions. Downstream metabolites, 3-phosphoglycerate and 2-phospho-glycerate were unchanged in the p73 KO mice, suggesting a distinct impact on glycolysis caused by loss of p73 expression (Figure 4). Upon starvation, the level of glucose in the liver dropped down in both groups of mice due to increased utilization by other organs. Interestingly, the levels of 3-phosphoglycerate and 2-phosphoglycerate were significantly reduced as compared to those of WT mice (Figure 4). Since both metabolites are also used to produce serine, which is the precursor of cysteine, low levels of those molecules, may be suggestive of limited availability of cysteine for glutathione synthesis in the p73 KO mice under starvation conditions. This again indicates that p73 mice probably suffer from oxidative stress.

**Figure 5:**
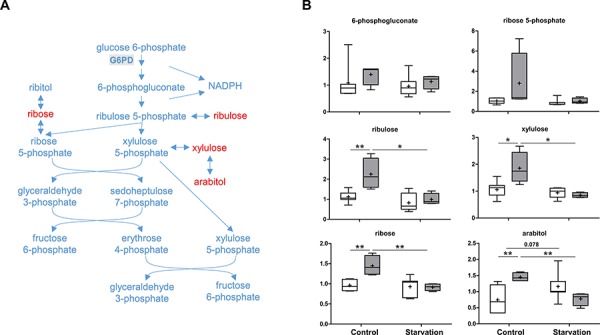
Pentose phosphate pathway (PPP) The PPP mainly produces NADPH and pentoses. As a rate-limiting enzyme, glucose-6-phosphate dehydrogenase (G6PD) performs the oxidation of glucose-6-phosphate, the first reaction of the PPP. **A.** A schematic diagram of the PPP. Levels of the metabolites found in this pathway are shown in **B.** G6PD: glucose-6-phosphate dehydrogenase. *P* values were calculated via 2-way ANOVA contrasts test. * < 0.05; ** < 0.01; *p* values between 0.05 and 0.1 are shown as numbers. *Y*-axis: relative amount of metabolite. Legends descriptions are the same as in Figure 2C. Metabolites showing significant changes are highlighted in red.

The second metabolite in glycolysis, glucose 6-phosphate can be oxidized by G6PD to enter the PPP (Figure 4 and Figure 5). Which pathway the glucose 6-phosphate enters is dependent on the current needs of the cell and on the concentration of NADP^+^ in the cytosol. In liver, the PPP is mainly used to metabolize excess glucose and to produce NADPH, allowing the maintenance of the antioxidant glutathione in its reduced form. TAp73 has been shown to enhance PPP activity and to support cell proliferation by transcriptional activation of G6PD, the rate-limiting enzyme of PPP [29]. Contrary to this report [29], and in agreement with our previous data [31, 32], loss of p73 expression did not reduce, but rather increased, PPP activity as evidenced by several upregulated end products, including arabitol, ribose, ribulose and xylulose under control conditions (Figure 5). This indicates that other p73 family members or additional transcription factors might also regulate the expression of G6PD. Nevertheless, these consistent elevated concentrations of end products point to the possibility of reduced hepatic glucose utilization or greater NADPH synthesis under non-starvation conditions when p73 is lacking. In addition, starvation induced a sharper decline in metabolites related to the PPP in p73 KO mouse livers compared to WT mouse livers. This finding might be related to increased PPP activity under control conditions, or, alternatively, to a differential carbohydrate metabolism when adapting to starvation in p73 KO mice. As mentioned above, an elevated PPP has important implications for the maintenance of glutathione in its reduced state, and, therefore, the response to oxidative stress.

### Alterations in citrate and krebs cycle intermediates in p73 KO mice

**Figure 6:**
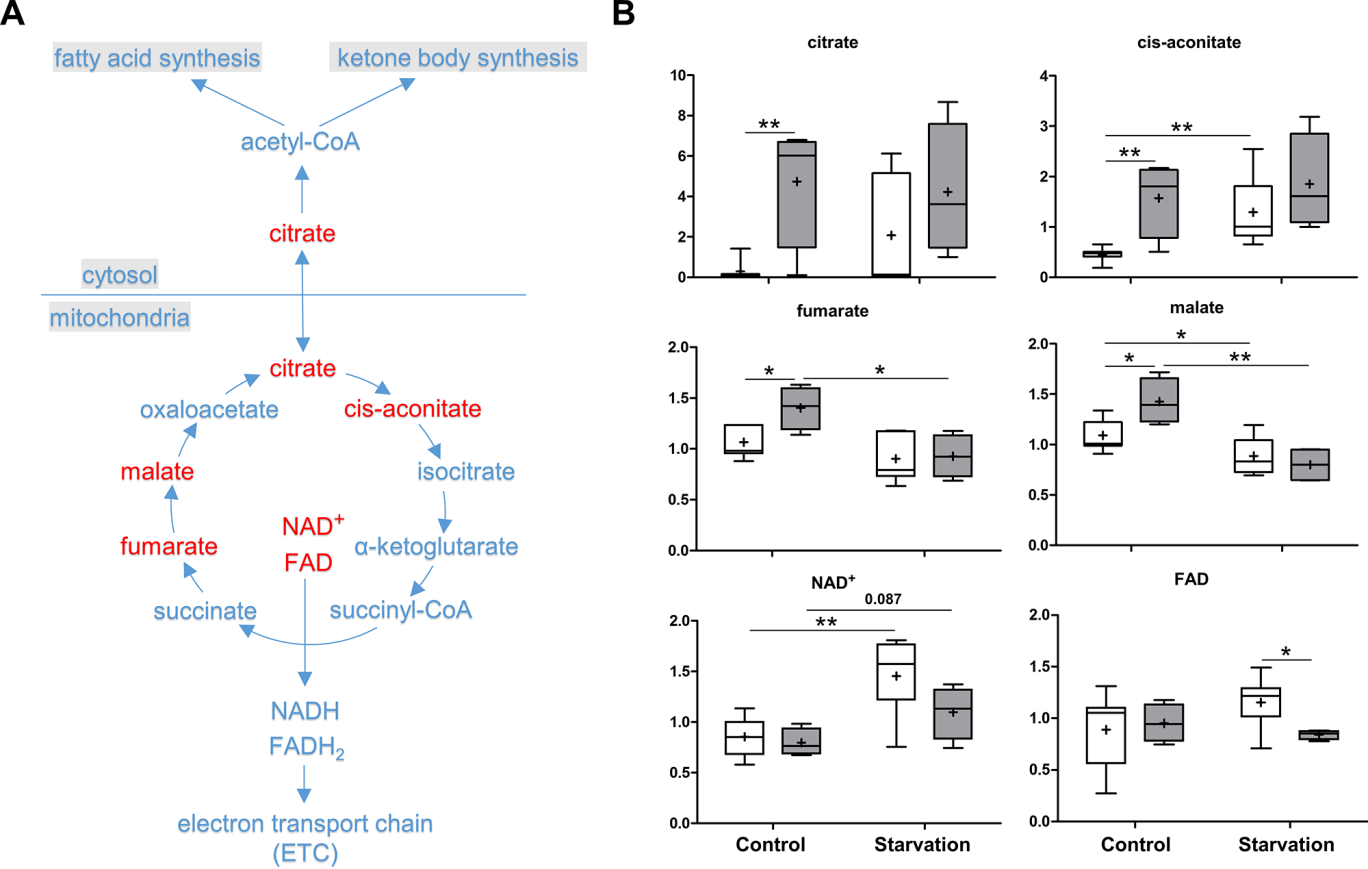
Krebs cycle and citrate shuttle The Krebs cycle is a core process for generating energy in the form of ATP in all aerobic organisms. The cyclic pathway begins with the formation of citrate, which can be also transferred from mitochondria into cytosol for fatty acid synthesis (citrate shuttle). **A.** A schematic diagram of the Krebs cycle and citrate shuttle pathways. Levels of the metabolites found in these pathways are shown in **B.** *P* values were calculated via 2-way ANOVA contrasts test. * < 0.05; ** < 0.01; *p* values between 0.05 and 0.1 are shown as numbers. *Y*-axis: relative amount of metabolite. Legends descriptions are the same as in Figure 2C. Metabolites showing significant changes are highlighted in red.

The Krebs cycle is a key component of cellular respiration for energy production. Several intermediates of the Krebs cycle were increased under control conditions when p73 is lacking, including citrate, cis-aconitate, fumarate, and malate. Notably, citrate showed more than 16 fold higher concentration in the p73 KO liver compared to WT. The increased concentrations of other compounds were not as pronounced as the increase of citrate (Figure 6). Changes in cycle activity in p73 KO livers under control conditions could explain these findings, although the directionality of change is difficult to predict from steady-state measurements. Alternatively, the changes could be due to shuttling of citrate from the mitochondria into the cytosol for fatty acid synthesis and, subsequently, incorporation into triglycerides. Importantly, a high concentration of cytosolic citrate allosterically inhibits phosphofructokinase-1, a rate-limiting enzyme in glycolysis, which may elucidate the abnormality in glycolysis and the PPP in p73 KO mice. Upon starvation, fumarate and malate were significantly reduced in both p73 KO and WT mice, probably due to an increased contribution to gluconeogenesis by oxaloacetate. In addition, the co-enzyme NAD+ was not significantly increased and the level of FAD was reduced in the p73 KO mice livers under starvation conditions (Figure 6). Since both metabolites are generated by the electron transport chain (ETC) for ATP production, these changes point to the possibility of reduced ETC activity in livers of p73 KO mice.

## DISCUSSION

While autophagy [36–40] has a particular relevance in cancer biology [41–43], several indications, already suggest that this relationship is affected by p53 and its family members [44–50]. Other indirect evidence also suggests the same conclusion [51]. For example, p73, which strongly affects neuronal [52–55] and cancer [56–59] biology, is regulated by its protein degradation by the E3 ubiquitin ligase Itch [60–63]. Interestingly, the inhibitors of Itch are indeed regulators of autophagy [64].

Our previous work reported that p73 regulates autophagy and lipid metabolism in hepatocytes via transcriptional activation of ATG5 [33]. Autophagy, a highly regulated self-digestion process, is kept at a low level to maintain cellular homeostasis. Dysregulated autophagy has been shown to be associated with neurodegenerative diseases, infections, and cancer [65–67]. Autophagy plays a key role in regulating cell survival under starvation conditions by increasing the availability of nutrients to maintain energy production [68, 69]. In the present study, starvation-induced metabolic changes were observed in livers of both WT and p73 KO mice. Some of the altered metabolic pathways could be the consequence of defective autophagy in liver cells of p73 KO mice. For instance, the generally lower levels of amino acids and the higher lipid content of p73 KO compared to WT mouse liver likely result from reduced autophagy [33]. Moreover, autophagy-regulated removal of damaged mitochondria could affect the cellular respiration system as well [70].

Based on our *in vivo* observations, p73, besides its role in autophagy, seems to directly affect distinct metabolic pathways, including amino acid metabolism, glycolysis, PPP, the response to oxidative stress, Krebs cycle, and fatty acids metabolism. In the metabolism of amino acids, it has been shown that TAp73 transcriptionally controls a key enzyme of glutaminolysis, GLS-2, that favors the conversion of glutamine in glutamate, which in turn promotes the serine biosynthetic pathway [25]. In line with this finding, the levels of glutamate and serine were found to be reduced in livers of p73 KO mice when compared with those of WT mice. Furthermore, upon starvation, lysine and methionine metabolism as well as glutathione synthesis were altered in the absence of p73 expression. As lysine degradation primarily occurs in the liver and the products of its degradation mainly contribute to production of ketone bodies, the elevation in these metabolic pathways in livers of p73 KO mice under starvation conditions suggests the usage of distinct fuels or a lack of energy derived from other metabolites. Related to the amino acid metabolism, altered glutathione synthesis could be observed in the p73 KO mice, as the alternative anti-oxidant ophthalmate was increased upon starvation, highlighting the potential role of p73 in oxidative stress responses.

Under certain conditions, glucose can be diverted to the PPP that is important for metabolism of excess glucose, for production of NADPH, and for synthesis of nucleotides. p53 can inhibit G6PD expression by direct interaction with G6PD or can promote G6PD expression by the transcriptional activation of its target, TIGAR, suggesting important roles of p53 family members in the PPP [71, 72]. Recently, it has been reported that TAp73 increases PPP flux to promote NADPH and ribose synthesis by transcriptional activation of a rate-limiting enzyme, the G6PD [29]. Moreover, these authors have also shown that depletion of TAp73 reduced the PPP flux, which could be rescued by G6PD expression. However, our study showed significant increases in glucose and several end products of PPP even when expression of all p73 isoforms was lacking under control conditions. In line with our finding, a more recent study showed no difference in the PPP and an increase in glycolysis in TAp73β-overexpressing Saos-2 cells [73]. These data suggest that the transcriptional control of G6PD is not uniquely regulated by TAp73; accordingly we suggested also a regulation by p63 [74]. Alternatively, these results may even indicate that p73 family members are able to inhibit the PPP, and hence act as tumor suppressors, in a similar manner as p53.

The significant increase in citrate and other intermediates of the Krebs cycle may be a consequence of changes in the cycle activity or, alternatively, be owing to citrate shuttling from mitochondria to the cytosol in p73 KO mice under control conditions. High level of citrate in the cytosol of hepatocytes promotes in turn fatty acid synthesis, inhibits glycolysis, leading to alternative glucose utilization by the PPP under control conditions, and, subsequently, contributes to ketogenesis under starvation conditions. Moreover, the lower levels of NAD^+^ and FAD in the p73 KO upon starvation also point to possible defects in the ETC, since the expression of a mitochondrial ETC subunit, cytochrome c oxidase subunit IV isoform 1 (Cox4i1), is under the control of TAp73 [23]. Notably, reduced ETC activity leads to reduced ATP levels, increased ROS production, and dependence on glucose, explaining the altered glutathione synthesis and distinct glucose utilization observed in the p73 KO mice. It is also important to mention that these processes are relevant autophagic targets [75–83]. Moreover, TAp73 isoforms most likely contribute to the regulation of metabolism owing to their transactivation domain. However, ΔNp73 isoforms might also be involved owing to their dominant-negative property. For instance, ΔNp73 isoforms might negatively regulate the expression of G6PD and, consequently, elevate PPP in p73 KO mice. Furthermore, the p73 isoforms generated by different C-terminal splicing events might participate in the metabolic regulation as well, since both TAp73 and ΔNp73 KO mice show less severe phenotypes compared to the total p73 KO mice [16–18].

Cancer cells often modify their metabolism by promoting diverse biosynthetic pathways to adapt to the environment and to ensure their rapid proliferation [84–92]. Besides aerobic glycolysis, the PPP is also activated to provide metabolites for nucleotide synthesis and NADPH to compensate the oxidative stress in cancer cells. As TAp73 is considered as a tumor suppressor, our work showing altered glycolysis and upregulated PPP further implies a metabolic role for p73 in tumor suppression. Furthermore, fasting has an anti-aging effect and can be used to help fighting against several diseases, including diabetes, hypertension, asthma and cancer [93]. p73 may therefore play a fundamental role in these starvation-induced processes, since p73 has also been shown to be involved in aging, inflammation, and tumorigenesis [23, 94]. Hence, follow-up studies focusing on the different specific pathways are recommended. Overall, our *in vivo* findings in livers showed that p73 depletion resulted in several altered metabolic pathways pointing to the importance of p73 in the basal and starvation-induced hepatocellular metabolism.

## MATERIALS AND METHODS

### Mice

p73 KO mice were generated as previously described [16]. Mice were bred and subjected to listed procedures under the Project Licence PPL 40/3442 released from the UK Home Office. The starvation experiments with the mice were performed by food deprivation for 24 hours with free access to drinking water. Liver samples from both WT and p73 KO mice were collected and immediately frozen for metabolic analysis.

### Metabolic analysis

Both WT and p73 KO mice were either fed ad libitum or starved for 24 hours. Liver samples from these mice were collected, immediately stored at −80°C until extracted and prepared for analysis using a standard solvent extraction method. Mass spec analysis was either performed in house at the MRC or at Metabolon^®^ (www.metabolon.com). The sample preparation process was carried out using the automated MicroLab STAR^®^ system (Hamilton Company). To purify the small molecules, the protein fraction was removed using an organic and aqueous extractions. The resulting extract was divided into two fractions, one for analysis by liquid chromatography (LC) and one for analysis by gas chromatography (GC). The organic solvent was removed by placing the samples briefly on a TurboVap^®^ (Zymark). Each sample was then frozen and dried under vacuum. After drying, samples were then prepared for the appropriate instrument, either LC/MS or GC/MS. After log transformation and imputation with minimum observed values for each group, the comparison of the metabolites of each sample was performed. The LC/MS portion of the platform was based on a Waters ACQUITY UPLC and a Thermo-Finnigan LTQ mass spectrometer, which consisted of an electrospray ionization (ESI) source and linear ion-trap (LIT) mass analyzer. The samples destined for GC/MS analysis were re-dried under vacuum desiccation for a minimum of 24 hours prior to being derivatized under dried nitrogen using bistrimethyl-silyl-triflouroacetamide (BSTFA). The GC column was 5% phenyl and the temperature ramp is from 40° to 300° C in a 16 minute period. Samples were analyzed on a Thermo-Finnigan Trace DSQ fast-scanning single-quadrupole mass spectrometer using electron impact ionization. Compounds were identified by comparison to library entries of purified standards or recurrent unknown entities. The quality control and curation processes were designed to ensure accurate and consistent identification of true chemical entities, and to remove those representing system artifacts, mis-assignments, and background noise. See [22, 31, 32, 74] for further details.

### Statistics

The metabolic analysis described above comprises 347 named compounds. Following 2-way ANOVA analysis, contrasts were used to identify metabolites that differed significantly between experimental groups. The total list of compounds that showed statistical significance (*p* < 0.05), as well as those approaching significance (0.05 < *P* < 0.10) were presented in Table 1. The *p* values for each compound, including P_contrasts_, P_genotype_, P_treatment_ and P_interaction_, are indicated in Supplementary Table S1.

## SUPPLEMENTARY FIGURES AND TABLES





## Abbreviations

ANOVA: analysis of variance
ATG5: autophagy-related protein 5
ATP: adenosine triphosphate
Cox4i1: cytochrome c oxidase subunit IV isoform 1
ETC: electron transport chain
FAD and FADH2: flavin adenine dinucleotide
G6PD: glucose 6-phosphate dehydrogenase
GC: gas chromatography
GLS-2: glutaminase-2
GSH: reduced glutathione
GSSG: glutathione disulfide
KO: knockout
LC: liquid chromatography
NAD+ and NADH: nicotinamide adenine dinucleotide
MS: Mass Spectrometry
NADP+ and NADPH: nicotinamide adenine dinucleotide phosphate
PPP: pentose phosphate pathway
WT: wild-type
