# Supplementary material for: p73 regulates basal and starvation-induced liver metabolism in vivo
Source: Oncotarget. 2015 Sep 7;6(32):33178–90. doi: 10.18632/oncotarget.5090 (PMC4741757; doi:10.18632/oncotarget.5090)
Supplement: Supplementary file 1 [file oncotarget-06-33178-s001.pdf]

SUPPLEMENTARY FIGURE AND TABLE

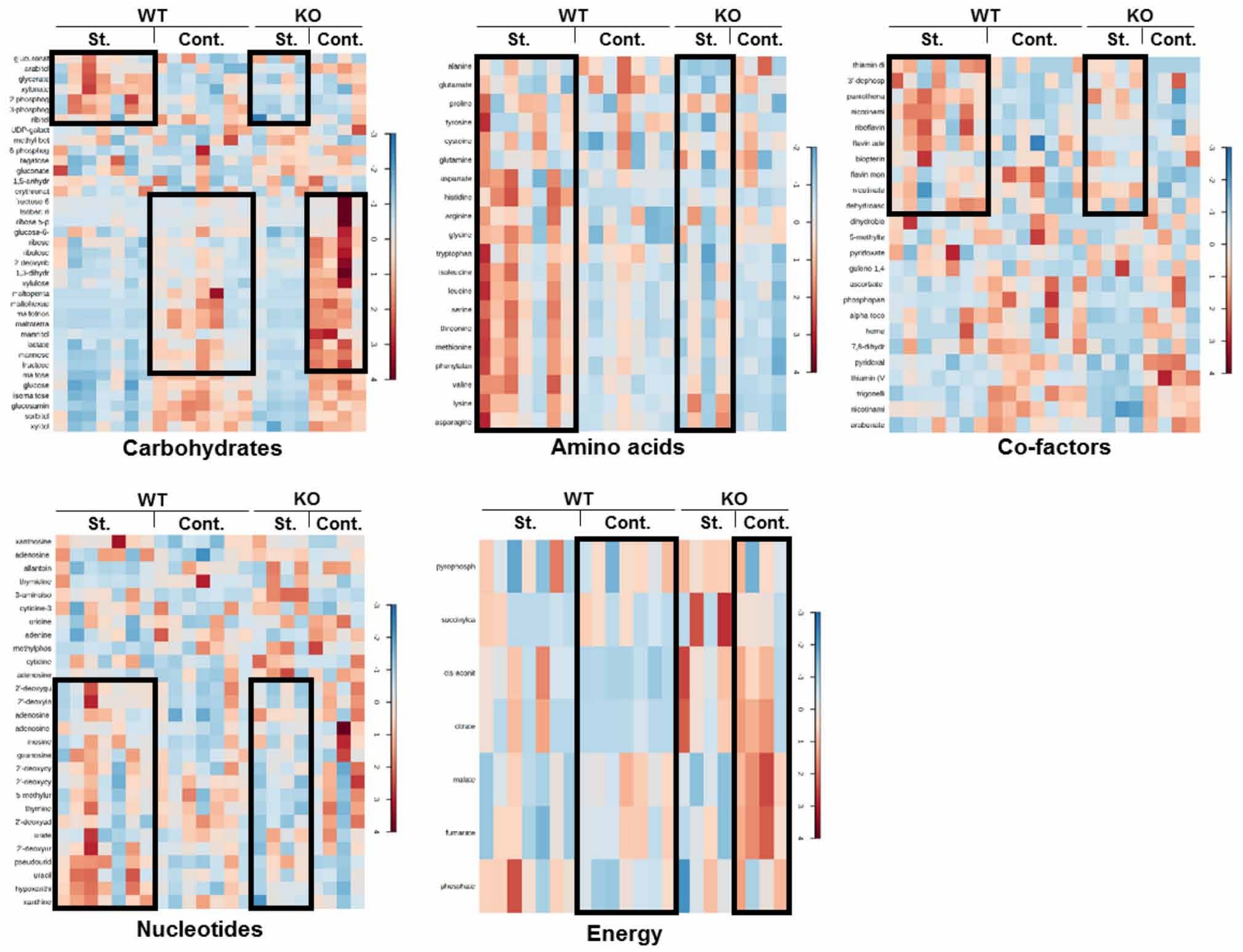

Supplementary Figure S1: Selected heat maps showing specific groups of metabolites. The black boxes indicate the extensive changes of the metabolites in different conditions. St.: starvation, Cont.: control.

Supplementary Table S1: Summary of 347 found metabolites. The *p* values of each compound including $P_{contrasts}$, $P_{genotype}$, $P_{treatment}$ and $P_{interaction}$ were calculated via 2-way ANOVA and ANOVA contrasts.
